# Sailing to save the planet? Media-produced narratives of Greta Thunberg’s trip to the UN Climate Summit in German print newspapers

**DOI:** 10.1057/s41599-023-01743-6

**Published:** 2023-05-15

**Authors:** Linda Lütkes, Leonie Tuitjer, Peter Dirksmeier

**Affiliations:** Leibniz Universität Hannover, Institute of Economic and Cultural Geography, Schneiderberg 50, 30167 Hanover, Germany

**Keywords:** Geography, Cultural and media studies, Environmental studies

## Abstract

Narratives and stories are important communication tools and as such essential subjects of social geography. This paper analyses the retelling of Greta Thunberg’s sailing trip across the Atlantic to the Climate Action Summit in New York in 2019 in leading German newspapers and magazines and how her intentions are transformed through this reporting into different narratives. The research mainly focuses on examining the influence of space and place, as geographical research has revealed that spatial determinants are crucial in risk communication and knowledge generation on climate change but have yet to be studied considering stories. The paper, therefore, extends the story-based approach from communication sciences by geographical research on the role of space and place in action-based stories. Therefore, the Narrative Policy Framework (NPF) is used to decode the spatial environment in narratives as an active element that shapes the narrative, and the way characters can (inter)act within these settings. The paper further develops the NPF framework through a geographical lens by focusing particularly on the selection options of spaces for social interactions and affective bonds. Thus, it becomes evident how spatial contexts and environments shape the interactions between individuals and crucially influence the types of narratives that emerge.

## Introduction

Since Greta Thunberg’s first school strike for the climate in August 2018, the global social movement ‘Fridays for Future’ has emerged in which Thunberg has been identified as a leading protagonist (Von Zabern and Tulloch, 2021). In Germany, some of the world’s largest and most widespread ‘Fridays for Future’ protests took place, setting climate change on the political agenda and giving climate protection a special significance in society and political discourse in recent years (Buzogány and Scherhaufer, 2022). Despite Germany being still one of the largest CO_2_ emitters in Europe, the then-governing German conservative party still justified coal production for economic reasons. Hence, the movement’s focus here was on the demand for a fast and consistent coal phase-out to achieve the goals of the Paris Agreement. Thunberg became a central figure in the movement in Germany and was known as the face of political resistance and climate activism (Bergmann and Ossewaarde, 2020).

In 2019, Thunberg attracted special attention in German media for her sailing trip across the Atlantic to the UN Climate Summit in New York. With this trip, which was described as climate-neutral, the young activist wanted to clarify her position on avoiding air travel and similar environmentally damaging behaviour and inspire people to become active in climate protection. Media and public interest in this trip were heightened by the fact that she was sailed by German star sailor and climate activist Boris Herrmann (Mkono et al., 2020). The analysis of the stories told about this journey in six German print newspapers is the subject of this study.

Research on climate communication has shown that media representations and especially stories can be effective tools for public understanding and risk perception of the climate emergency (Bevan et al., 2020). Cameron (2012, p. 574) points out: “Stories express something irreducibly particular and personal, and yet they can be received as expressions of broader social and political context, and their telling can move, affect, and produce collectivities.” Therefore, it is important to consider the performativity of stories; although there is an objective reality, people assign different meanings to the world around them and construct reality with the help of stories and narratives. These social constructions of the world and resulting social relations are influenced by values, norms, and beliefs (Schlaufer et al., 2022). To create effective climate communication and engage the public in climate action, it is proposed to tell more action-oriented stories to stimulate social learning and generate agency (Meyer et al., 2021). This paper aims to connect the story-based approach of communication studies with geographic research and explores the role of place and space in stories. Previous geographic research has shown that spatial determinants are important factors in risk perception, production of knowledge about climate change, and willingness to act (Devine-Wright, 2013; Mahony and Hulme, 2018; Rudiak-Gould, 2013). We will extend this approach by examining the role of place and space in action-based storytelling. We are interested in how narratives are created through them, and how spaces influence and shape characters’ actions. Moreover, our paper can counteract the lack of discussion on the role of space and place in narrative studies, which has been criticised in geography scholarship (Prince, 2018).

For this analysis, we rely on the Narrative Policy Framework (NPF), which serves as an analytical tool for decoding the beliefs, values, and content of stories by referring to typical structural elements of stories—characters, setting, plot, and moral (Fløttum and Gjerstad, 2017; Jones and McBeth, 2010). The systematic analysis of narrative elements in stories allows us to examine what factors initiate individual and collective action and have implications for political beliefs and agency. In particular, focusing on the element of setting in climate stories provides an opportunity to examine the influence of spatial determinants (Jones et al., 2014). Considering narrative structure and narrative context with the NPF distinguishes this approach from common approaches in human geography, such as discourse analysis (e.g. Foucault, 1972; Laclau and Mouffe, 1985).

Accordingly, we assume that the NPF provides a valuable approach to the geographical study of the role of place and space in climate stories. We exemplify this by analysing the four entangled aspects of character, setting, plot, and moral that shape the narrative around Thunberg’s journey, piloted by Herrmann. We emphasise the need for a geographical engagement with the setting of her journey, as spatial references can foster a sense of closeness and hence contribute to people’s engagement with climate change (Bloomfield and Manktelow, 2021). Special interest is given to the spatiality of the boat, which is shaped by the material limitation of space due to the narrowness of the ship and the constantly changing location far from the mainland. This creates a changed situational context and a special kind of place that can shape people’s lives and their relations to each other (Cresswell, 2004). This spatiality warrants a human geographic examination with questions about proximity and distance (Hasty and Peters, 2012), borders (Newman, 2006), and the very specific situational stratification in the two-week continuous interaction situation of Thunberg and Herrmann (Collins, 2000).

The paper proceeds as follows. First, we demonstrate the relevance of stories in climate communication and illustrate the role of space and place in risk perception and knowledge production. The NPF is a useful approach for climate communication strategies, as it allows for an analysis that shows which narrative elements can have an impact on individual or collective action and political processes. We then present our empirical material and methodology before analysing how different print media sources in Germany used the entangled elements of character, setting, plot, and moral within their reporting. A geographic sensibility within an NPF context enables us to understand how particular narratives and moral messages are formed that foreground the importance of a narrative approach to climate change communication research in geography for encouraging people to test alternatives and engage in climate action.

## Changing approaches in climate change communication

Many people perceive the scientific evidence of the climate emergency as abstract, distant, and impersonal, which makes it difficult to raise interest in and concern for the issue (Gustafson et al., 2020; Manzo and Padfield, 2016). Climate scientists and policymakers often struggle to communicate the content and consequences of climate change and to mobilise people for climate action (Bevan et al., 2020). Therefore, a central question in climate research is how climate communication can be made more effective and which methods can encourage people to become socially and politically engaged (Morris et al., 2019; Stoknes, 2014). To find answers to these questions, geographers have explored the role of climate communication in the form of media reports, stories, maps, and images. They have investigated how attention and concern about climate change are generated (Harris, 2020; Meyer et al., 2021) and how this is influenced by spatial determinants (Devine-Wright, 2013; Mahony and Hulme, 2018; Rudiak-Gould, 2013). These analyses focus primarily on how communication must be designed to convince people of the existence and risks of climate change. Currently, however, there are calls for a shift in the focus of climate communication to show the relevance of action and participation in policy changes (De Wit and Haines, 2022). Communication through stories is seen as an essential tool to influence individual and collective behaviour and shape public discourses (Cameron, 2012; Meyer et al., 2021). Although research shows that space and place play a critical role in communicating the causes and consequences of climate change, risk perception, and personal concern (Devine-Wright, 2013; Mahony and Hulme, 2018; Rudiak-Gould, 2013), spatial determinants in stories promoting climate change engagement have not been widely studied. This paper uses the Narrative Policy Framework to fill this gap and analyse geographical determinants in stories about climate change. This approach can be used as an analytical tool to clarify how (political) narratives influence individual and public opinion, which beliefs, values, and moral messages are conveyed, and which significance spatial conditions have (Fløttum and Gjerstad, 2017; Jones and McBeth, 2010). This approach thus combines geographical studies of the role of space and place in the communication of climate knowledge with the relevance of stories noted by communication scholars.

### Storytelling for climate action

Effectively addressing the challenges of climate change requires that people not only believe in climate change and understand the risks and impacts but also change their behaviour and take climate action (Meyer et al., 2021). The communication of scientific findings and demonstration of the impacts of climate change has led to the existence and risks of anthropogenic climate change becoming a widespread consensus, and society is increasingly concerned. Nevertheless, even among people concerned about the impacts of climate change, individual behaviour and commitment to social and political change remain low (Stoknes, 2014). Therefore, Morris et al. (2019) conclude that fact-based information presentations do little to motivate action and call for alternative approaches in climate communication.

Since the ‘narrative turn’, social science scholars have assumed that stories can be part of the solution (Veland et al., 2018) and “that stories have a fundamental role to play in our collective ability to address the complex realities of climate change” (Harris, 2020, p. 310). Stories follow a basic narrative structure consisting of a beginning, middle, and ending. They portray diverse characters and convey a moral message (Morris et al., 2019). By telling personal, character-based stories, it is possible to recreate concrete and personal experiences, provide insights into the characters’ thoughts and feelings, and thereby reduce psychological distance. This experiential transportation allows a stronger imagination and emotional connection to the characters and is considered helpful for communicating with diverse audiences, including non-expert audiences (Gustafson et al., 2020). Stories are seen as a way to bring together abstract, disconnected phenomena and thereby construct order and a view of the world. Veland et al. (2018) note: “In this way, narratives constitute reality as we know it by making sense of observations, leading us to new inferences, and providing models for a path forward” (p. 42).

In addition, it is considered important to tell diverse stories about people who are actively engaging in climate action and contributing to transformative change. Action-based stories stimulate social learning processes and can help recipients build agency for similar situations (Meyer et al., 2021). Meyer et al. (2021) therefore, see a shift in the “conceptualization of climate change from ‘issue-based’ to ‘action-based’” (p. 1) stories as crucial for effective climate communication. Learning from other people’s actions and experiences (Howarth and Parsons, 2021) is a way to overcome the lack of agency by developing “new, concrete ways of ‘knowing’ how to act in relation to climate change” (Meyer et al., 2021, p. 5). Furthermore, the research addresses the inclusion of celebrities in stories about climate change as their privileged voices can raise awareness, serve as role models, and trigger appropriate social action. Through celebrity activism, climate change could be perceived as less abstract and impersonal and gain media attention. Conversely, there is concern that celebrity involvement leads to a focus on the individual, obscuring politically complex issues and hindering change (Boykoff et al., 2010).

Communicating climate knowledge and opportunities for action through stories is not a substitute for presenting scientific facts but should be seen as a second, complementary path to contribute to understanding and motivation for action in a different way (Dahlstrom, 2014). Although the relevance of stories for climate change communication is already well-founded “little attention [has been paid] to spatial relations in narrative” (Prince, 2018, p. 175).

### The spatiality of climate communication

Place and space play an important role in climate communication for several reasons, such as addressing the visibility and localisation of climate change impacts (Brace and Geoghegan, 2011; Rudiak-Gould, 2013), contextualising knowledge and information (Mahony and Hulme, 2018), or incorporating identity, place attachment, and responsibility (Schweizer et al., 2013). To address the spatiality of climate communication, it is necessary to understand and distinguish between space and place. We will draw on the geographical discussion of these terms, as they are central concepts in this discipline (Cresswell, 2004).

Space can be understood as a more abstract concept of the physical area that can be described factually and mathematically, e.g., with the help of coordinates, sizes, and distances. Conversely, place refers to a location that acquires meaning through human interaction, social relationships, and cultural, social, and symbolic attachment. Meaningless space can therefore become a place through human action. Place as a meaningful location contains three dimensions—location, locale, and sense of place (Agnew, 2011). With ‘place’ a specific geographical reference is established, the place is located in space and set in relation to other places. Conversely, in the dimension ‘locale’, the place does not necessarily have to be associated with a specific location and does not always have to be spatially bound (Agnew, 2011). Cresswell (2004) notes: “A ship, for instance, may become a special kind of place for people who share it on a long voyage, even though its location is constantly changing. By ‘local’ Agnew means the material setting for social relations—the actual shape of place within which people conduct their lives as individuals, as men or women, as white or black, straight or gay” (p. 15). The local is thus not only an administrative unit and a physical boundary but also a place for accumulating interpersonal relationships and creating identity. At the same time, the local can also be used for representation, in which external influences and circumstances are spatially located and processed (Pfaff-Czarnecka, 2005). The third dimension, the ‘sense of place’, is the identification with a place that arises from subjective and emotional attachment. “In this construction, every place is particular and, thus, singular“ (Agnew, 2011, p. 327). In climate communication, the terms ‘space’ and ‘place’ are therefore relevant, as climate change affects both physical space and meaningful social place and manifests itself differently locally. To understand which spatial, social, cultural, and symbolic factors influence perception, knowledge, and action about climate change, it is therefore important to consider spatiality in climate communication.

Global climate change and global warming are not identifiable in their entirety through personal experience due to their complexity and spatial extent. Scientists, therefore, try to make climate change visible to the public with the help of climate models and risk projections (Rudiak-Gould, 2013). This statistical construction of climate change creates a global, universal body of knowledge that summarizes locally observed changes over time and transfers them to the global scale (Mahony and Hulme, 2018). This scientific knowledge about climate change is communicated through various mass media, political, and professional conferences, and, most importantly, in the form of scientific assessments, such as the Intergovernmental Panel on Climate Change reports. This is intended to create a common consensus on the facts of climate change, justify the need for adaptation measures, and initiate change (Hulme, 2008). However, it is criticised that this knowledge, presented as global and universal, does not do justice to the different knowledge claims around the world and that more attention should be paid to spatial differences. For example, place-based knowledge from vulnerable indigenous communities is not considered sufficiently in the scientific construction of climate change, although climate change is already most noticeable in these places (Mahony and Hulme, 2018). Moreover, Hulme (2008) notes that “by constructing climate change as a global problem, one that is distanced and unsituated relative to an individual’s mental world, we make it easy for citizens to verbalise superficial concern with the problem, but a concern belied by little enthusiasm for behavioural change” (p. 8). Scannell and Gifford (2013) suggest that greater attention should also be paid to the role of place in climate communication. Local, personal, and current relevant messages will reduce the abstractness of the phenomenon, arouse personal concern, and promote engagement (Devine-Wright, 2013). This is attributed to psychological distance, i.e., the more distant a phenomenon is in the four dimensions of “social, spatial, temporal, and outcome certainty” (Rudiak-Gould, 2013, p. 66), the more difficult it becomes to imagine. Therefore, a close attachment to place and nature can lead to the strengthening of pro-environmental behaviour and engagement, and to the emergence of sociocultural embedded knowledge that meets local demands (Devine-Wright, 2013). Locally occurring impacts of climate change can also be used to communicate the risks of climate change across space and to socially construct climate change elsewhere. For example, local individual phenomena, such as images of melting glaciers, stories of sinking islands, or voices from vulnerable communities, are used to highlight the wider and global issues of climate change (Rudiak-Gould, 2013).

Accordingly, geographic research has explored the role of place in climate change knowledge production and risk perception and has shown that place-based, personal narratives can help communicate climate change in culturally accessible ways. Meanwhile, less research has explored the role of space and place in stories that are less focused on communicating risk and more concerned with inspiring climate action and highlighting opportunities for social and political change.

### Narrative Policy Framework for geographical research

The geographical interest in narratives and stories has increased since the ‘narrative turn’ in social sciences (Cameron, 2012; Godioli, 2018). It has become clear that geographical locations and space play a crucial role in stories, for example, creating social and cultural expectations, contributing to emotional identification with the characters, or enabling the contextualisation of the story (Hyvärinen, 2018). In addition, the narrative form of stories enables readers to experience space and place emotionally and see spaces from other perspectives (Parker, 2018). This is justified, for example, by “links between plot progression and movement across spaces” (Prince, 2018, p. 175). Moreover, it became clear that a division can be made between the place where the story is told and the space in which the associated discourse can be located (Prince, 2018).

Although discourse analysis is typically used in geography (e.g. Farbotko and Lazrus, 2012; McEvoy and Wilder, 2012) to analyse the role of place and space in public and media discourses, this study relies on an alternative approach to analyse spatial determinants in action-based climate stories—the Narrative Policy Framework. The focus on stories and the narratives they produce justifies the NPF because it approaches discourse from a perspective that follows the storyline, the characters, and their intentions and allows for a spatial focus by looking at the setting. In this way, fixed elements and narrative strategies in stories can be identified, and thus, discourses can be empirically operationalised (Jones et al., 2014; Jones and McBeth, 2010; Jones and Peterson, 2017). The NPF is built on four crucial elements—characters, settings, plot, and morality (Fløttum and Gjerstad, 2017; Jones and McBeth, 2010).

NPF theory postulates that (political) narratives contain different characters, at least heroes, villains, and victims who act in specific locations, and the setting (Fløttum and Gjerstad, 2017; Jones et al., 2014). The hero figure is of particular relevance. If sufficient opportunities for identification are created, this triggers personal involvement on the reader’s part. Gustafson et al. (2020) point out that the experiences of the story characters lead to a better empathy with the problems and thus, possibilities for action become visible. The setting, as the second element of NPF theory, refers to a moment when the political problem is played out and forms the contextual framework (Jones et al., 2014). Local settings and spatial reference points create personal references and perspectives that are more likely to be familiar to the audience, which further increases the potential for identification (Bloomfield and Manktelow, 2021). By telling stories with a sense of place and local interests and highlighting possibilities for action there, these stories contribute to local knowledge and promote the ability to act. In this way, approaches to climate change adaptation can be better integrated into local social and cultural life (Ayeb-Karlsson and Uy, 2022; Howarth and Parsons, 2021). It is here where geographic research and concepts become particularly relevant and communication studies and geography can mutually enrich each other. Third, a typical story contains a beginning, a middle, and an end. The relationship between the different characters is shown along this plot line; the events are placed in spatial and temporal contexts and located in the setting (Fløttum and Gjerstad, 2017). The fourth and decisive element is the moral of the story—this should show and exemplify possible political but also personal solutions and ideas for action (Bevan et al., 2020). The NPF can be used to successfully investigate the role of place and space in climate change stories, as it is explicitly adapted to narrative structure and content (Bevan et al., 2020). In addition, the approach lends itself to a focus on action-based climate narratives, as NPF is used to examine the influence of narratives on individual and collective action. The NPF is useful for identifying narrative elements, such as the setting, that have implications for political beliefs and agency (Jones and McBeth, 2010).

Research shows that communicating facts about the risks of climate change is not enough to move people to action and engage them in social and political change. Action-based climate stories are needed to persuade people, stimulate social learning, and motivate them to take climate action. Geographic research shows that place, space, and place-based identity are critical to successful climate communication. The role these elements play in action-based climate stories is explored using the NPF as a theoretical lens, representing a new approach in geographic research.

## Qualitative NFP research design

Gray and Jones (2016) present a guide for applying the NPF in qualitative research, which consists of four steps and forms the basis for this analysis. The first step involves describing the policy issue and contextualizing it. In our case, this is the analysis of Thunberg’s trip to the UN Climate Summit 2019. The research design is developed in the second step, and the methodology is selected. In the third step, the data is analysed, and the analysis process is discussed, followed by the presentation of results in the last and fourth step.

### The policy issue

The research object of the study is Thunberg’s journey across the Atlantic to the UN Climate Summit 2019 in New York. Her intention can be summarised as drawing attention to the climate emergency and highlighting the possibilities and limitations of climate-friendly travel (Mkono et al., 2020).

Thunberg’s voyage has attracted much attention in the German print media, which has been enhanced by the prominence of her co-sailor Herrmann. Looking at the media coverage, her voyage is reproduced as a story: The young climate activist Thunberg, in the role of the main character, is invited to the UN Climate Summit in New York and crosses the Atlantic on Herrmann’s zero-emission racing yacht “Malizia”. The sailing crew, consisting of Thunberg and her father, professional sailor Herrmann, co-skipper Pierre Casiragh, and a cameraman, started on August 14, 2019, in Plymouth, United Kingdom, sailed more than 3700 nautical miles and reached New York within 14 days. On her way, Thunberg is exposed to the dangers of the ocean, must deal with the lack of comfort on-board the yacht, and earns both admiration and criticism for the trip.

This journey has triggered a controversial discussion in the German media, which tells different stories, portrays the characters in different lights, and draws different moral lessons from the journey. Moreover, an interesting aspect of media portrayal is the importance of the story’s setting. The following analysis will examine how the setting, and thus the role of place and space, influences the defining narratives and narrative elements in the story of Thunberg’s journey.

### Data collection

We analyse the corresponding narratives of her journey in six German print newspapers: *Frankfurter Allgemeine Zeitung* (*F.A.Z*.), *die Tageszeitung* (*taz*), *Die Zeit*, *Stern*, *Hamburger Abendblatt* and *Nordwest-Zeitung*. These newspapers cover a broad political spectrum. We compare different types of newspapers and establish a spatial reference to Herrmann’s origin. *F.A.Z*. (conservative-liberal) and *taz* (left-alternative) are national daily newspapers. *Die Zeit* is a liberal political weekly based in Hamburg. *Stern* is a weekly political ‘general-interest newspaper’ (Pürer and Raabe, 2007). The regional newspapers *Hamburger Abendblatt* and *Nordwest-Zeitung* (published in Oldenburg) refer to the place of residence (Hamburg) and birthplace (Oldenburg) of Herrmann, respectively.

Considering the representativeness of these selections, the *F.A.Z*., with around one million readers per issue, has the third-largest reach of the national daily newspapers in Germany. Only the tabloid-newspaper *BILD* and the left-liberal newspaper *Süddeutsche Zeitung* have a larger readership. The *taz* reaches around 300,000 readers per issue, placing it sixth among Germany’s national newspapers. *Stern* and *Die Zeit* are two of the 17 weekly newspapers in Germany that report on current political events (Agma (2023)). In addition, regional newspapers were included in the analysis because they reach 41.3 percent of the population in Germany and are thus the most widespread type of newspaper. The selection of the two newspapers is justified by their spatial relevance (Agma, 2020).

The period of analysis starts with Thunberg’s Twitter post on June 17, 2019, that she had decided to accept the invitation to the UN Climate Summit 2019 and was looking for a way to travel emission-free across the Atlantic. It ends on August 30, 2019, two days after Thunberg’s arrival in New York. Relevant newspaper articles were compiled with the help of the WISO and LexisNexis databases and the *F.A.Z*. online archive. Under the search term “Greta Thunberg”, a total of 49 articles were found in the six newspapers that dealt with Thunberg’s journey.

Methodologically, qualitative content analysis is used (Hsieh and Shannon, 2005; Mayring, 2004; Neuendorf, 2017; Schreier, 2013). The central component of the method is the rule-guided assignment of text passages to previously defined categories, which are derived from a combination of deductive and inductive elements (Flick, 2013; Mayring, 2004).

### Qualitative NPF data analysis

Four main categories of NPF theory (characters, plot, moral, and setting), presented, for example, by Jones and McBeth (2010), guide the analysis of the story of Thunberg’s Atlantic crossing. These four elements form the deductive basis for the four main categories of the code, for each of which subcategories were formed. For the category ‘characters’ represent the four roles of hero, mentor, victim, and villain given by NPF; the plot consists of the categories beginning, middle, and end, while the moral was divided into positive, neutral, and negative coverage of Thunberg’s media-effective protest action. Finally, the setting was divided in terms of spatial reference aspects that create either proximity, distance or symbolic meaning. These codes were included in a coding guide and provided with corresponding coding rules.

The analysis was conducted by the research team using the qualitative analysis software Atlas.TI and followed the guidelines for qualitative content analysis according to Mayring (2004). The newspaper articles were analysed using the coding guide and the defined categories were assigned to specific text passages. In the pilot phase, the categories and codes were reviewed and adjusted. After the analysis was completed, an intracoder analysis was conducted in which part of the material was coded again and both versions were compared with each other. The validity of the coding is also supported by the multi-authorship as the codes were developed by the research team and the results of the coding were discussed afterwards. This ensures the reliability of the coding process. To achieve the research goal of examining the role of space and place in action-oriented climate narratives, the presentation of findings focuses on the setting. The other narrative elements influence and shape the setting, or are shaped by the setting, and thus occupy important parts of the findings.

## Spatiality in the narratives of Thunberg’s sailing trip to New York

**Fig. 1:**
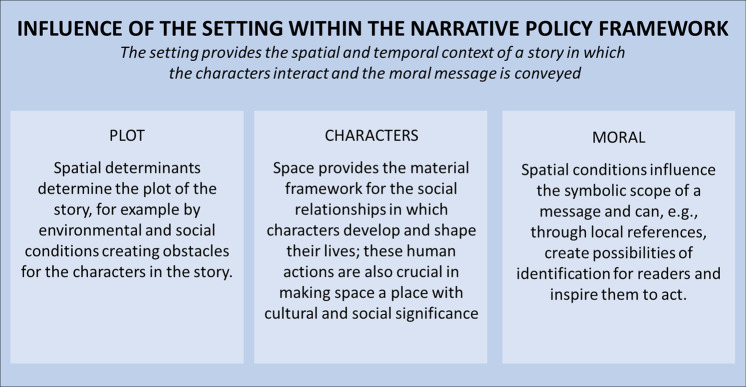
Influence of the setting within the Narrative Policy Framework. The figure shows the role of setting within the Narrative Policy Framework and its influence on the other elements—plot, characters, and moral (own illustration by Linda Lütkes, no permission required).

The main objective is to examine the role of place and space in action-based stories of climate change, using the example of Thunberg’s sailing trip to the UN Climate Summit 2019. Thunberg’s motive for her trip is to raise awareness of the climate emergency and to show the possibilities and limits of climate-friendly travel. In the following, the Narrative Policy Framework is used to analyse the role of the setting in the story and how spatial factors influence the plot, the characters, and their social interactions. Furthermore, the role of spatiality and locality in the moral message of the story and thus in the possibility to inspire action and engagement for social and political change will be analysed. Therefore, the physical space and the social meaning of the location (place) with local references are relevant for the analysis. In Fig. 1, it is shown that the setting and spatial determinants influence the other structural elements of the Narrative Policy Framework.

### Spatial determinants shape the storyline

The plot of a story, consisting of a beginning, middle, and end, situates the characters in time and space and establishes relationships between the characters and their environment (Jones and McBeth, 2010). Setting plays a crucial role in this process, as it helps, for example, to create conflicts and obstacles that the characters must overcome (Weible and Schlager, 2014). Therefore, an analysis of the setting is carried out to understand how space and place can shape the plot of the story of Thunberg’s transatlantic crossing.

The media narrative begins with the young climate activist being invited to the UN Climate Summit in New York and her wish to attend it. The spatial distance, the crossing of the ocean, is the first obstacle the protagonist has to overcome on her journey, as she wants to avoid flying by plane for environmental reasons. The *F.A.Z*. writes: “In June, Thunberg had set out via Twitter to find the most emission-free means of transportation possible to get from Europe to the United States” (Reuter, 2019, p. 7, own transl.). Space is thus an important factor to overcome by Thunberg to achieve her goal. With her journey, she wants to show that “the world can also be changed by individual behaviour, and it is an appeal to the global community for more action against climate change” (Kreutzfeld, 2019, p. 3, own transl.).

In Herrmann’s racing yacht ‘Malizia’, Thunberg finds a climate-neutral way to cross the Atlantic. The yacht then becomes the spatial setting for the main part of the story. The sailing ship is described as an “uncompromisingly designed racing yacht” (Rüdiger, 2019, p. 26, own transl.), as a “racing yacht is never comfortable, but offers only the most minimal equipment in the pursuit of lightness. […] There are no toilets on board, and instead of beds, there are only two bunk beds” (DPA, 2019, p. 6, own transl.). The crossing of the ocean by sailing ship is described in the newspapers as a danger and challenge for the climate activist and the sailing crew to overcome to get to New York. This is shown by Herrmann’s statements quoted by the *Hamburger Abendblatt*: “The North Atlantic is rough; we have big unpleasant waves in case of doubt. In addition, there are strong winds and, above all, the fight against sea sickness” (Laufer, 2019, p. 11, own transl.). On the one hand, this creates a spatial distance linguistically, describing the vastness of the sea and the uncontrollability associated with it. However, at the same time, it also shows spatial proximity, for example, in the closeness to nature, since, for example, the travel plans are crucially dependent on weather conditions and the forces of nature. The *Nordwest-Zeitung*, therefore, considers “the Malizia II as an extremely fitting ship for Thunberg and her commitment to climate protection. The Fridays for Future-movement deserves credit for bringing a simple but uncomfortable fact back to society’s attention: We are dependent on nature even if we often suppress this in our highly technological everyday life” (Kleingärtner, 2019, p. 4, own transl.).

The sea as a spatial obstacle on the way to New York can be perceived not only as a physical distance but also as a border between social spaces with different cultures. The boat is seen as a means to overcome this border (Hasty and Peters, 2012). However, these boundaries can have not only divisive but also unifying effects by fostering a sense of belonging and togetherness and enabling differentiation from others. Especially in the context of globalisation and the facilitated crossing of borders, their property of ‘creating order’ has become more important (Newman, 2006). Thunberg’s way of travelling, sailing across the ocean in a yacht, deviates from the conventional mobility of the globalised world. Instead of crossing the Atlantic by plane within a few hours and taking advantage of globalisation’s simplified border crossing, Thunberg takes an uncomfortable, two-week journey. “The 16-year-old travels by yacht because planes emit large amounts of greenhouse gases—and because there is no direct train connection from her hometown of Stockholm to the US metropolis” comments the *Hamburger Abendblatt* (Barth, 2019, p. 21, own transl.). The fact that travelling by yacht is nowadays not perceived as a suitable way of travelling is also reflected in the lower status that the ship has in mobility research compared to the car, train, and airplane (Hasty and Peters, 2012).

In the end, Thunberg and the sailing crew overcome the dangers of the ocean and reach New York. 17 ships welcome the ‘Malizia’ in New York to symbolise the Sustainable Development Goals. Fans of Thunberg awaited the sailing crew. Locations play an important role in the plot of a story because of their ability to have a symbolic effect (Huxford, 2007). This can be recognised in the figurative effect of New York created in the reporting by using emotionally charged images. On the one hand, it is described as a place of freedom and unlimited possibilities, reflecting, for example, Herrmann’s statement quoted in the *F.A.Z*. “‘We will dock our yacht in New York directly in Manhattan below the Freedom Tower, where the World Trade Center once stood,’ says the 38-year-old German professional sailor. ‘That will be a very special atmosphere’” (Reuter, 2019, p. 7, own transl.). At the same time, New York is pictured as being representative of America and as “delicate terrain” (Lindner, 2019, p. 9, own transl.) for Thunberg, since “Donald Trump, after all, is not exactly considered an energetic fighter against climate change, as he signalled to the whole world at the latest by pulling out of the Paris climate agreement” (Lindner, 2019, p. 9, own transl.). On the one hand, New York can be seen as a political space from which Thunberg can distance herself, and at the same time, through the diversity and openness of the city, find like-minded people and develop a sense of belonging (Newman, 2006).

The analysis of the spatial determinants in the story’s plot shows that the setting, consisting of space and place, can have an important influence on the narrative of Thunberg’s journey. The perception of physical space and distance changes through the means of transport and the travellers themselves, and the physical space develops into a place that decisively shapes the plot of the story, in which the roles of the characters develop and influence their relationships with each other.

### Space becoming place

Space and social interactions are intimately related in stories—while space provides the material setting for social connections in which characters develop and shape their lives, these human actions are also crucial for turning space into a place with cultural and social significance (Cresswell, 2004). This can also be observed in the story of Thunberg’s journey.

Throughout most of the story, the spatial setting is strongly limited by the boat as it serves as a means of transportation, a place to live and sleep and interact with each other. Although it constantly changes its geographical location, it becomes a special kind of place for the five passengers of the boat (Cresswell, 2004). The ship is also perceived as a special means of transportation, characterised by the double burden of proximity (to fellow passengers and crew) and isolation (from the mainland) as well as the proximity to nature with all its dangers (Hasty and Peters, 2012). The isolation from the mainland and being cut off from the ordinary world causes a decoupling from society and a new form of social reality, reflected in the perception or role assignment of fellow passengers.

Thunberg is perceived on land as the celebrity of the voyage, admired even by the professional sailor Herrmann. Thunberg is described positively in all the newspapers studied with phrases such as “climate pop star” (Rüdiger, 2019, p. 26, own transl.), “perhaps the world’s most famous teenager” (Plickert and Reuter, 2019, p. 7, own transl.), and “the face of a global movement” (Schwinghammer, 2019, p. 3, own transl.). Thunberg is hence perceived as heroic and iconic, a courageous young climate activist. Through this reporting on Thunberg and her actions, she helps alleviate feelings of hopelessness and helplessness related to the threat of climate change (Mkono et al., 2020). This embodiment of role models by celebrities is important for increasing interest in environmental issues and can have a mobilising effect (Hanna et al., 2018). Furthermore, the description of Thunberg as a heroine allows readers to empathise with her role and to follow more abstract information about climate change (Fløttum and Gjerstad, 2017; Gustafson et al., 2020).

Thunberg is also portrayed as a role model for Herrmann. The sailor’s quote in the *Hamburger Abendblatt* underlines his admiration for the young climate activist: “She stands up against ignorance and injustice in relation to the climate crisis with amazing courage. I feel humble that Greta has accepted our offer to cross the Atlantic on the ‘Malizia’ as the cleanest and most environmentally friendly option—despite the lack of comfort for her” (Hasse, 2019, p. 11, own transl.). Herrmann himself is also a celebrity away from the voyage, celebrated in Germany as a professional sailor, and the newspaper *taz* assumed that “at least in terms of publicity, Thunberg’s voyage should also benefit from Herrmann” (Hansen, 2019, p. 2, own transl.). In the context of the story, Herrmann can be associated with the role of a mentor figure. By offering to sail Thunberg across the Atlantic, he gives her the opportunity of a low-emission Atlantic crossing and supports her in achieving her goals.

As the setting changes, the roles and social interactions between the characters also change. The boat can therefore be understood as a place of an alternative order, where the normal conventions of identity are subverted (Hasty and Peters, 2012). The spatial setting for example reinforces Herrmann’s position as the mentor, clearly putting Thunberg into the position of the novice and helpless passenger whilst allowing for the portrayal of Herrmann as the leading figure during the trip. Thunberg puts herself in his hands and trusts his technical skills, while he is trying to do everything for her well-being while at the same time, he must tame the forces of nature on the ocean alone, for example, by “sailing on gentleness” (Widmann, 2019, p. 1, own transl.). He also pays for the costs of the trip. Despite Thunberg’s fame, she is the subordinated figure during the voyage and furthermore takes on the role of the guest who is in an intermediary position between stranger and friend for a limited period of time (Pitt-Rivers, 1968). Thunberg, as a guest, does not have to work but can “send pictures of impressive amounts of water to the world” (Widmann, 2019, p. 1, own transl.). In contrast, the *F.A.Z*. uses the setting to form Thunberg’s victim role. This ship is not perceived as a suitable place for people with Asperger’s syndrome and Thunberg is put in this uncomfortable situation by her father as the quote exemplifies: “And does this father know what he is doing to his daughter and to himself with this trip? People with Asperger’s syndrome are used to fixed habits and rituals, have difficulties with social communication and motor problems” (Breymann, 2019, p. 21, own transl.). The newspaper *Stern* shows the effects of isolation from Thunberg’s point of view. “Honestly, I’m really looking forward to finally being isolated. Just to have nothing else to do. No interviews. No obligations. And also not so much internet and cell phone” (Breng and Stwaski, 2019, p. 28, own transl.). Isolation strengthens Thunberg’s capacity for self-determination and can therefore be distinguished from the role of the victim.

The alternative order on board the ship shows that all social phenomena depend on the situational context in which they occur. Collins (2000) speaks of situational stratification because the usual social positions in society are not accessible at that moment, and interaction takes place in a way detached from society. This particular situation and the altered interaction between the two celebrities lasts two weeks. Although Thunberg’s and Herrmann’s interaction is disconnected from direct influence from the outside world, the event is simultaneously perceived and judged through worldwide news coverage. Michel de Certeau (1988) illustrates this with the metaphor of ships as dungeons. According to de Certeau, ships form closed systems in which the passengers are at the mercy of complete surveillance, and there are no possibilities of escape. Nevertheless, this spatial separateness also forms the possibility to distance oneself from reality, act separately, and to take on new roles. At the end of the journey and when leaving the ship, the passengers return to the roles in society that belong to them (De Certeau, 1988). During the voyage, Herrmann mainly assesses whether the boat is on course and when an arrival can be expected. But the moment the boat arrives in New York and the symbolic ‘door of the dungeon’ is opened, former role relations are restored. Thunberg is cheered by her fans, is received as a guest at the climate summit, and is thus the centre of attention. The *F.A.Z*. reported in detail on the reception committee that welcomed Thunberg in New York. “She was greeted by loud ‘Greta! Greta!’ shouts, some singing ‘Welcome, Greta’ to the tune of ‘Brother John’. Several hundred people have come, including many children and young people, as well as a huge media contingent. It is a handsome group, though not a mass gathering. People hold banners with inscriptions like ‘Make America Greta Again’ or ‘Welcome to New York’” (Lindner, 2019, p. 9, own transl.). For Herrmann, on the other hand, the journey and accompanying media attention ends here and he returns to his everyday life. “Boris Herrmann flies back, and trees are planted to compensate for the CO_2_ emitted. And four members of his team want to attempt a world record and chase the Malizia back to Europe in seven days. And immediately afterward, they will train in the Bretagne for the great adventure”, writes *Die Zeit* (Widmann, 2019, S. 1).

### Setting as a sense of place

The setting also plays an important role in conveying the moral message, with spatial factors influencing how messages are articulated and received and whether they can inspire engagement (Jones and McBeth, 2010). The moral of the story, as an important component for pointing out possible solutions and encouraging action, shows on the one hand that the reporting conveys Thunberg’s motive for action, to draw attention to the climate emergency with her journey and to show the possibilities and limits of climate-friendly action (Mkono et al., 2020). This becomes obvious in the report in the *Hamburger Abendblatt*: “Greta has to travel climate neutrally as a symbolic figure of a movement and thus show how it might be possible” (Heider, 2019, p. 2, own transl.). In the *F.A.Z*., forecasts are made on what impact such symbolic effects will have on climate protection. “Assuming (only) 10 million people really liked the campaign and decided to save one kilogram of CO_2_ once. In purely arithmetical terms, that would be 10,000 metric tons, which is probably at least a hundred times what could theoretically have been saved. Doesn’t this prospect alone justify such an action?” (Reichel, 2019, p. 18, own transl.). The unusual way of overcoming spatial distance by ship thus illustrates the limits and possibilities of climate-friendly action in the globalised world. The *taz* underlines this symbolic component of the journey: “But by getting into a sailboat and travelling unusually, she prolongs the attention for the urgency of climate policy to a maximum. Let’s put it simply: Greta Thunberg’s sailing trip to America could become the most important since Christopher Columbus” (Unfried, 2019, p. 2, own transl.).

However, the reporting is also characterised by negative associations with Thunberg’s journey, questioning and criticising her actions. For example, the *taz* published that several transatlantic flights were necessary to realise Thunberg’s trip, as the sailing crew travelled back to Europe by air. The report says: “Greta’s sailing trip is more harmful to the climate than flying; Greta Thunberg’s trip to the USA on a yacht is emission-free. But more Atlantic flights are needed to bring the boat and crew back than if she had flown herself” (Maurin, 2019, p. 12, own transl.). An additional critique is that crossing the Atlantic by racing yacht does not illustrate any possibility of action for the normal citizen, because it is not feasible in terms of time and money. The *taz* writes: “There is no climate-friendly option for ordinary people these days. After all, only extremely few sailors cross the Atlantic. A two-week tour is simply too long for most people with a normal vacation and time allotment. And ocean sailors also have little room for passengers. Cargo ships take only a few guests, and even then, only for hefty fees of, for example, more than 1,000 Euros each way” (Maurin, 2019, p. 12, own transl.). This option is also not an alternative for other participants of the UN Climate Summit. For example, the *taz* comments, “Greta’s sailing trip cannot necessarily be a model for Angela Merkel. Sailing takes time, and sponsorship is certainly not an option for a chancellor” (Barth, 2019, p. 21, own transl.). Accordingly, no direct possibility of action is shown, and a distance is created between Thunberg and the readers’ lifeworld. In contrast to what climate storytelling is aiming for, this does not highlight any options for action that could encourage climate-friendly agency but rather conveys a mood of despair and hopelessness (Meyer et al., 2021).

The dedicatedly leftist newspaper *taz* is most critical of the trip^1^. Here there is the least sympathy for the extraordinary effort of crossing the ocean by boat and the most critical research into the extended carbon footprint of her voyage. The *taz* assumes that climate-friendly lifestyle changes cannot be achieved by conveying symbolic messages alone. It is crucial that concrete, realistic alternatives for action are demonstrated. “That’s why it would have been good, very good if Greta Thunberg had appeared at the climate summit via monitor. The message: You don’t all have to fly all the time to achieve what you want. Sometimes a conference call is enough. True, it’s much more boring than travelling by racing yacht. But it can have a much greater impact in the long term. Because then the question is whether a trip is necessary at all. Sometimes the journey is not the destination after all” (Gaus, 2019, p. 2, own transl.). The dimension of morality is here understood as a policy recommendation, in line with NPF theory (Fløttum and Gjerstad, 2017). Pointing out realistic options for action is considered decisive in climate communication, as only such options are adapted to everyday behaviour and accepted by citizens (Meyer et al., 2021). Therefore, it must be critically discussed whether overcoming the physical space in the globalised world through a sailing trip across the Atlantic is necessary to make people aware of the climate emergency through media attention. The approach taken by the *taz*, overcoming great distances by technical, digital means in the form of video conferences, is shown as an alternative for action. The UN Climate Summit and the coverage of Thunberg’s trip already took place in the summer of 2019, before the Covid-19 pandemic, when communication via video feeds in such contexts was much less popular. It becomes clear that moral messages, in various ways, can be found in the news discourse. However, another article in the same newspaper confirms Thunberg’s decision to change location and attend the climate conference in person:

“Greta Thunberg refers to a different sense of travel, a different sense of life, and a different understanding of politics. She is about using her time for what is important to her, that is the moral of this story. So state, European, and transnational climate politics. That’s why she travels to New York to speak there. Because she has to be there if she wants to influence the actors there, the event, and its media reception. You can’t do that with a Skype call” (Unfried, 2019, p. 2, own translation).

In addition to overcoming space to symbolise the message, local references also play a role in how the message is communicated to the readers. The reporting makes spatial reference to Herrmann’s origins and home. While the nationally published *F.A.Z*. creates identification possibilities for people from different places with expressions such as ‘German professional sailor’, ‘Hamburger’ or ‘Native of Oldenburg’, a focus on the respective city can be seen in the regional newspapers. In the reporting by *Nordwest-Zeitung*, spatial proximity is created by references to Herrmann’s childhood in Oldenburg, for example by mentioning that he learned to sail on Lake Zwischenahn in the Oldenburg region. The *Hamburger Abendblatt* reinforces the sense of belonging to Hamburg as his place of residence by mentioning his everyday life, his life partner living in Hamburg, and his involvement with Hamburg Climate Week. Previous research has demonstrated the importance of localities as places of belonging, as a social environment, and as platforms for identification (Antonsich, 2010; Bloomfield and Manktelow, 2021). Local attachment can contribute to forming identity narratives and, thus, constitutes an important element of stories (Tomaney, 2015). Stories being told in the context of a sense of place and local interests, and by highlighting possibilities for action, can contribute to local knowledge. Stories promote the ability to act at this level. As a result, approaches to climate change adaptation can be better integrated into local social and cultural life (Howarth and Parsons, 2021).

Space and place play an important role in conveying the moral message in the story of Thunberg’s journey to the UN Climate Summit. On the one hand, overcoming space with the unusual means of transport of the ship is crucial for the symbolic scope of the message and the accompanying media attention. On the other hand, local references can also trigger a sense of belonging and build a possibility of identification with the characters (Agnew, 2011). Spatial determinants thus shape the way the message is constructed and how it is conveyed and received.

## Conclusion

This paper shows the relevance of storytelling in climate communication to highlight possibilities for action and to convince people to engage in social and political change processes. Although it has already been studied that space and place are important factors in communicating climate emergency, it is criticised that the role of spatial determinants in climate change narratives has not yet been widely studied. Using the re-narrating of Thunberg’s Atlantic crossing on a racing yacht piloted by German professional sailor Herrmann to attend the UN Climate Summit in New York as an example, the paper analyses how stories are influenced by space and place. The analysis of six German newspapers, selected to represent a broad section of the political spectrum and various newspaper types (weeklies, dailies, regional newspapers), is based on the Narrative Policy Framework. We especially focused on the role of geographical settings (the boat, the sea, New York, and the place attachments of Herrmann as a German citizen who lives in Hamburg), highlighting that setting as a spatial context is not only a passive background on which the storyline unfolds but an active element that shapes the narrative and the ways characters can (inter)act within these settings. From a geographical perspective, we especially explored the ambivalence of proximity and distance, the spatiality of the boat, and the affordances and constraints the boat has for social interactions.

We advance the NPF with a geographic lens by particularly focusing on the constraints and affordances of spaces for social interactions and affective attachments. In addition, we foreground that spatial contexts and environments influence the other structural elements of the Narrative Policy Framework—characters, plot, and moral—thus decisively influencing the types of emerging narratives.

In our example, it becomes clear that space and spatial distances change in the context of the attempt to travel in a climate-friendly way and become central obstacles for Thunberg as the protagonist, thus decisively determining the storyline. The perception of physical space is therefore dependent on the characters who act in it. The characters are shaped by the setting that surrounds them, defining their roles and shaping their social interactions. This becomes clear, for example, through the setting of the boat, which, despite the constantly changing geographical locality, develops into a special place for Thunberg, Herrmann, and the crew and redefines their social roles. The setting also influences the construction and communication of the moral of the story. The unusual way of overcoming the physical distance of the ocean—by sailing yacht instead of by plane, as is common in the globalised world—symbolises the moral message of the story and provides media attention for the climate emergency. At the same time, local references, for example to Herrmann’s origins and place of residence, create opportunities to identify with the story and its characters, to take action, and to engage in social and political change processes.

Our paper hence not only contributes novel empirical insights but also experiments with the NPF to take spatiality and settings as central foci in how we narrate stories about climate change and actions to combat it. We conclude this paper by highlighting the need for greater consideration of geographic determinants in action-oriented narratives in climate change communication to inspire engagement and a sense of efficacy in people.

## Data Availability

All newspaper articles used in the analysis are publicly available.

## Footnotes

1 Parties that support climate and environmental protection and movements like Fridays for Future are typically located on the political left in Germany (Oswald et al., 2021). Thus, it is surprising that a left-wing, green-oriented newspaper is most strongly opposed to Thunberg’s trip.
